# Conspicuous Coloration in Males of the Damselfly *Nehalennia irene* (Zygoptera: Coenagrionidae): Do Males Signal Their Unprofitability to Other Males?

**DOI:** 10.1371/journal.pone.0142684

**Published:** 2015-11-20

**Authors:** Christopher D. Beatty, José A. Andrés, Thomas N. Sherratt

**Affiliations:** 1 Department of Biology, Santa Clara University, 500 El Camino Real, Santa Clara, CA, 95053–0268, United States of America; 2 Department of Ecology & Evolutionary Biology, Cornell University, E149 Corson Hall, 215 Tower Road, Ithaca, NY, 08053, United States of America; 3 Department of Biology, University of Saskatchewan, Saskatoon, Saskatechewan, S7N 0W0, Canada; 4 Department of Biology, Carleton University, 1125 Colonel By Drive, Ottawa, ON, K1S 5B6, Canada; CNRS, FRANCE

## Abstract

In damselflies, sexual colour dimorphism is commonly explained as a consequence of selection on traits that increase male attractiveness to females. However, while many species in the damselfly family Coenagrionidae (Insecta: Odonata) are sexually dimorphic, the males do not engage in displays, and male competition for mates resembles a “scramble”. An alternative explanation for the sexual differences in coloration within these species is that sexual dimorphism has evolved as a sex-related warning signal, with males signalling their uprofitability as mates to other males, thereby avoiding harassment from conspecifics. We evaluated an underlying assumption of the theory that male-male harassment rate is influenced by colour by comparing harassment of males of the species *Nehalennia irene* that had been painted to make them appear: (i) similar to an unaltered male (blue), (ii) different from a male (orange) and (iii) more similar to a female (black). When caged together we found that blue-painted males experienced significantly lower harassment than black-painted males. When unpainted males were caged with each type of painted male we found that blue-painted males and the unpainted males housed in the same cages experienced lower rates of harassment than males housed in cages where some males were painted black, suggesting that a single, reliable signal of unprofitability may benefit the individuals that carry it. While our results do not in themselves demonstrate that sexual colour dimorphism originally evolved as an intra-specific warning signal, they do show that harassment is influenced by coloration, and that such selection could conceivably maintain male coloration as a warning signal.

## Introduction

Many damselfly species demonstrate sexual colour dimorphism in which males are brightly coloured, while females are more drab [1–3]. Traditionally, conspicuous male coloration has been attributed to premating sexual selection acting on male characters: that is, competition among males to gain access to females, with males actively displaying their quality as mates, either in their physical attributes or in the quality of the resources that they defend [4–11]. Among many species in the family Coenagrionidae, however, males do not defend territories or engage in displays to females [3]. In these species, mating resembles a “scramble” with males searching for mating opportunities and pursuing potential mates aggressively, at times coercing them to mate. Furthermore, while males in these species are conspicuously coloured, the colour patterns are displayed primarily on the dorsal surface of the male body, rather than over the entire surface of the body or on their wings. In mating damselflies, males use claspers at the distal end of their abdomen to attach to plates on the thorax of the female; to receive sperm, females then bring their abdomen forward to attach to the male genetalia located on the ventral side of the second abdominal segment; this is often referred to as the ‘wheel’ formation [12]. Males approach females from above as they prepare to go into tandem with them, and as such, their coloration may not be immediately visible to the female.

So, what selective forces are behind the evolution and maintenance of bright coloration in males of this group? Sherratt and Forbes [13] suggested anti-harassment aposematism as an explanation; specifically that bright coloration has evolved in the males of these damselfly species as a form of sex-related warning coloration. Through verbal and quantitative models they demonstrated that if: (a) male-male interactions are costly (mediated through direct energetic/physical costs and/or indirect opportunity costs), and (b) males can potentially utilize coloration to differentiate males from females, then sexual dimorphism with a conspicuously colored male form and a cryptic female form is likely to evolve. In effect, distinctive male coloration may have evolved by selection as an “identifcation badge” to avoid confusion in the scramble for mates and thereby minimize the costs of erroneous mate selection. Sherratt and Forbes [13] argued that even under conditions of increased predation of males due to increased conspicuousness to predators, sexual dimorphism could still arise if the costs of harassing (or being harassed) by individuals of the same sex were sufficiently high.

Other researchers have also suggested potential examples of intra-specific warning signals. Papaj and Newsom [14] found that the coloration of larvae of the pipevine swallowtail, *Battus philenor*, deterred conspecific females from ovipositing on an already occupied host plant. Indeed, Poulton [15] offered a similar example in his original definition of “aposematism”, highlighting the case of an *Ichnuemon* wasp that avoids laying its eggs on host larvae if it detects the bright colors of the eggs of a conspecific.

While Sherratt and Forbes’ model suggests that male conspicuousness could have evolved to avoid male-male harassment, no study has tested the validity of this idea in the field. While it is difficult to demonstrate through experiments whether the conspicuous coloration of males initially evolved as an aposematic signal, the assumption that this selective force could maintain conspicuousness as a signal of “unprofitability” can be tested using behavioural experiments. A key prediction of the intraspecific aposematism hypothesis [13] is that if coloration indeed serves as an indicator of sexual identity to other males, then individuals showing the male coloration should be harassed less than individuals with coloration more similar to that of the female. In this paper we test this prediction by examining whether males of the species *Nehalennia irene* (Odonata: Coenagrionidae) receive significantly different harassment from other males when their coloration is altered.

## Materials and Methods

### Ethics Statement

All experiments performed comply with the laws for animal handling and field observations for Canada and the province of Ontario. These experiments were performed at localities on the property of Queen’s University Biological Station (QUBS); experimental design and property access were approved by QUBS management. *N*. *irene* is not a protected or endangered species in Ontario.

### Study organism

*N*. *irene* is one of the most abundant and widespread damselflies in Ontario [16]. Adults are small in size (approximately 26–28 mm in length). Males are dark in colour with metallic blue-green markings and with a conspicuous area of blue containing a pair of black spots on the dorsal side of abdominal segments 9 and 10. Females in this region demonstrate a colour polymorphism, with a drab pale green form (commonly referred to as a ‘gynomorph’ or ‘heteromorph’) and a male-like (‘andromorph’) form [17–19]. Male damselflies of this species and others, will often engage in ‘face-off’ behaviour when approached by other males, orienting themselved head-on to the other male [12].

In Ontario the adult flight season of *N*. *irene* lasts from approximately May 30^th^ through August 20^th^. *N*. *irene* is found predominantly in still water habitats, in marshy and boggy ponds, streams and temporary bog pools [16].

### Experimental design: general considerations

Previous studies of male response to coloration predominantly used dead (*i*.*e*. pinned) individuals and artificial models [20] or live individuals attached to a perch or tether [21,22,23]. While informative, the conclusions of these studies are limited, as the response of the focal males is known to be the result of both the colour and behaviour of the models [22,24]. To avoid this potential bias, we performed all behavioural experiments in semi-natural conditions using outdoor experimental insectaries (1.8m × 1.8m × 1.8m Bioquip Outdoor Cages, 32 × 32 thread count Mesh Lumite netting (product #1406C), tan colour) with natural vegetation and water features as oviposition substrate (see Study site/experimental setup). All individuals in our study were active (though there was variation in the level of activity among individuals) and quite readily approached other individuals and attempted to mate. Ovipositions were observed in the water features placed in the cages, thus indicating that experimental conditions were favorable to completion of the mating sequence.

**Fig 1:**
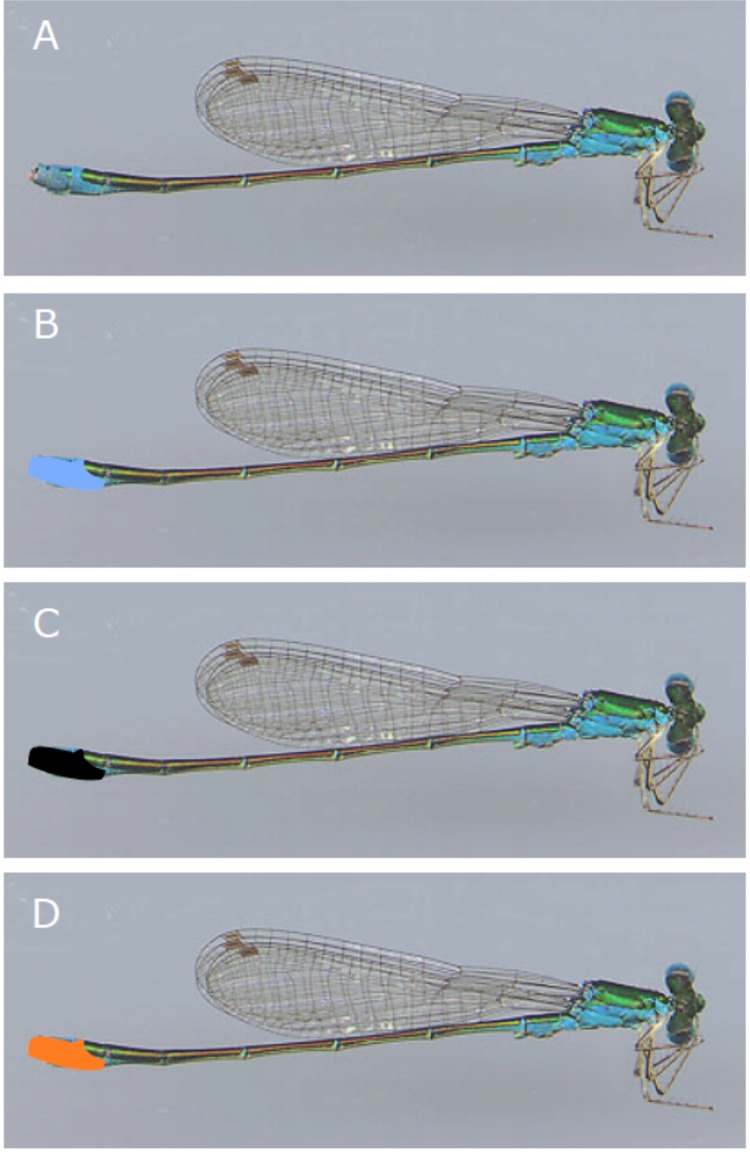
Colour manipulation of males of *Nehalennia irene*. Shown are scans of an individual male; A) shows an unaltered indivual, while B-D) show images marked with blue, orange and black in the areas altered with paint in these experiments.

To test for differential harassment of manipulated colour forms of *N*. *irene*, we altered male coloration by painting the dorsal side of abdominal segments 9 and 10 with three different colors of non-toxic paint (all paints made by Ultra Gloss acrylic enamel, DecoArt, Stanford, KY; the three colours used were: DG28, ‘country blue’, DG07, ‘orange’ and DG02, ‘gloss black’). Similar paints have been used successfully in other studies of female damselfly colour and behavior [25]. Abdominal segments 9 and 10 are bright blue in males and andromorphic females of *N*. *irene* while much of the rest of the abdomen is black in color (Fig 1A); heteromorphic females of *N*. *irene* lack this blue abdominal coloration, with a black dorsum on the end of the abdomen. While there are other colour differences between males and gynomorphic females in *N*. *irene*—males also have blue patterning on the thorax that females lack, and gynomorphic females are pale yellow on the ventral side of the abdomen, while males are dark—the conspicuous blue coloration at the distal end of the abdomen is an obvious component of the phenotype, especially when viewed from above, where the dorsal yellow of gynomorphs is less obvious. While painting the dosal side of the male abdomen does not cause males to be perfect female mimics, a change in the colour of this relatively small portion of the body makes a large difference in the overall appearance of individuals. Previous studies of male response to female (and male) coloration [20,21] have determined that abdominal coloration is the major component used in mate recognition by males.

Altered males in our experiments were painted one of three colors: blue, such that they continued to appear similar to males (Fig 1B), orange, an entirely novel colour in the species (Fig 1C), and black, making males appear similar to females, at least to human eyes (Fig 1D). Male *N*. *irene* should be able to perceive orange colour, as a long-wavelength photoreceptor appears to be ancestral in damselflies [26]. This experimental design does not account for any ultraviolet (UV) component to coloration. Odonates are known to have visual systems capable of detecting light in the UV range [27–29]. So, it is theoretically possible for UV to be an important component of odonate coloration. However, while colour studies of male coenagrionid damselflies have found some minimal reflectance in the UV range [30,31] the spectra of these odonates showed the highest percentage reflectance at wavelengths above 400nm, in the human visible range. Therefore our focus on altering visible coloration seems appropriate to assess the behavioural implications of conspicuous coloration in males.

All the individuals used for our experiments were collected each morning between 8:45 and 10:30 local time with sweep nets and placed in holding cages (1m^3^), sorted by gender. At 10:30 males and females were removed from the holding cages, painted, and placed in the outdoor experimental insectaries. While all males of each colour in a cage were painted together, the order in which paint colors were applied to males was randomized among cages. The order in which cages were loaded was also randomized each day. All females included in the experiments were gynomorphic (heteromorphic), as these are the majority morph in the populations under study, and our focus was the influence of potential harassment on males as predicted by the intraspecific aposematism hypothesis.

### Study site/experimental setup

This research was performed at Queen’s University Biological Station, approximately 50 km north of Kingston, Ontario, Canada. The research site, known as Barb’s Marsh (44°31’30”N, 76°22’20”) is an approximately 3.7 hectare permanent beaver pond surrounded by open meadows. Outdoor insectaries were assembled approximately 3m from the wet margins of the pond, in an area where the vegetation had been cut to approximately 10cm in height. For experiment 1 (2004), four cages were set up in a 2 × 2 grid, with a one meter gap between the cages. In experiment 2 (2005), 3 cages were set up in a straight line parallel to the shore of the pond. A 4L water container was placed in the southwest and northeast corners of each cage, with garden canes placed in the container to serve as perches. Cages were seeded with small flying insects collected with sweep nets from the surrounding field to maintain a supply of prey items for the damselflies. Ambient air temperature and wind-speed readings taken inside and outside the cages indicated that the interior cage conditions were similar to exterior conditions; the interior of the cages were approximately 2–3 °C cooler than ambient temperatures each day (ambient temperatures ranging from 21–27 °C during the course of the experiments), and wind speeds were slightly diminished due to the cage material (0–3 km/hr).

**Fig 2:**
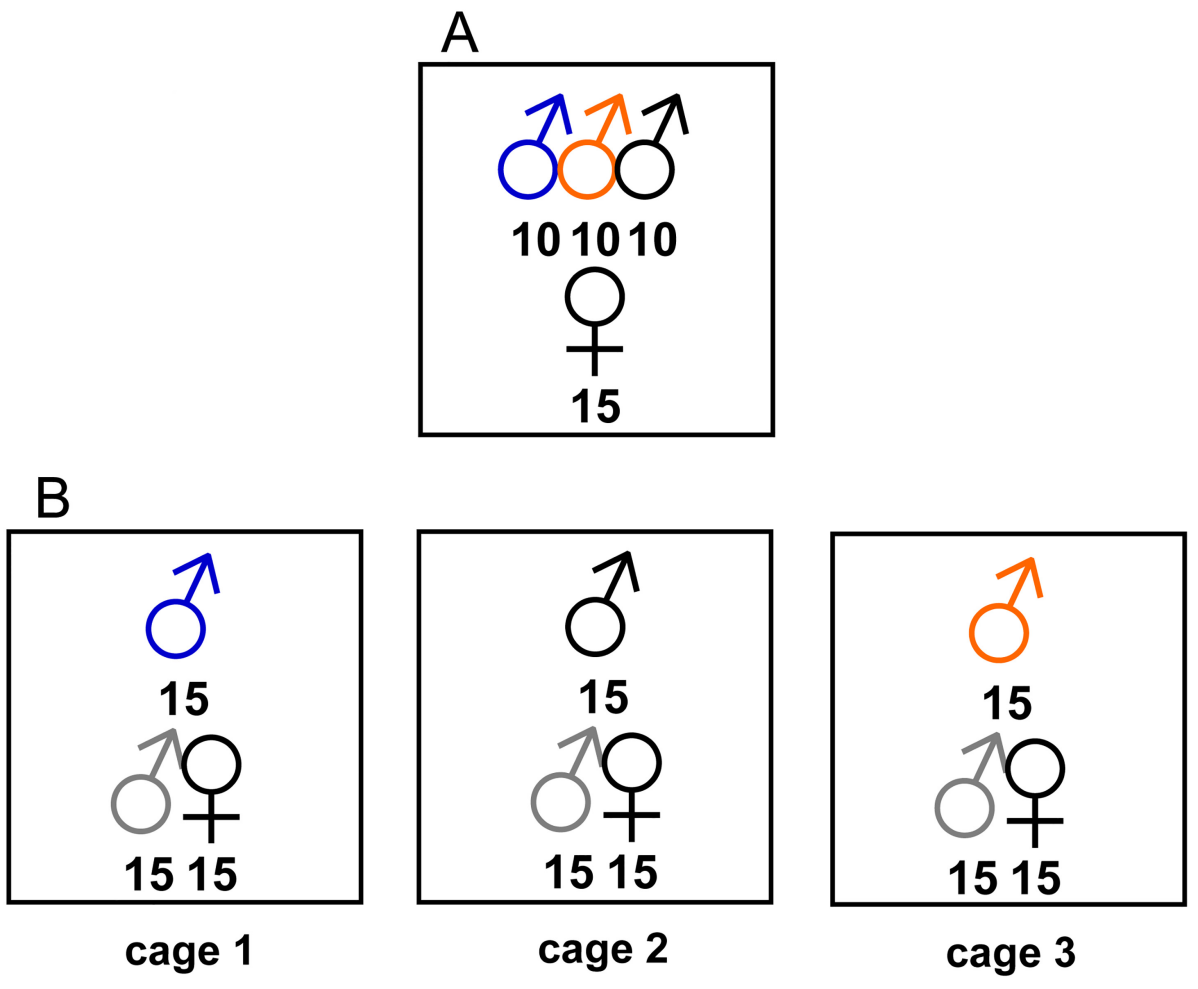
Design for intraspecific aposematism experiments with *Nehalennia irene*. A) Design for experiment 1. Each cage contained 10 males of each painted color and 15 females. B) Randomised-block design for experiment 2. Each cage contained 15 painted males, with a different paint color applied in each of the three cages. Each cage also contained 15 unpainted males (shown adjoining females) and 15 females.

We conducted two different experiments. In experiment 1, 10 males of each colour (blue, orange and black) were placed in each of the four cages along with 15 females (see Fig 2A). Total sex ratios were 2:1 for males to females, with a density of approximately 13 individuals/m^2^. In these environments near the edge of the pond, sex ratios are normally male biased, often at ratios even higher than those generated here; densities in this experiment are high compared to natural densities, which may range from 5–10 individuals/m^2^, but are not out of line with densities during peak daily activity. This experiment allowed us to directly compare the behaviour of individuals of each colour type. A total of 5 trials (with four identical cages per trial for a total of N = 20 cage replicates) were run between June 23^rd^ and July 4^th^, 2004 on sunny/partly cloudy days.

Between 12:30 and 16:00 local time on the day that each experiment was begun, an observer sat in the corner of each cage for 30 minutes, recording each harassment between individuals. A harassment was recorded if one individual perched on top of another individual and attempted to form a tandem. The giver and receiver of harassment in each interaction were recorded: whether the individual was male or female, and if male what colour it was painted.

In experiment 2, 15 males painted one of the three colours were placed in a cage with 15 unpainted males (all males were handled in the same way; unaltered males were brushed with a dry paintbrush before being introduced to the cage) and 15 heteromorphic females (identical density and sex ratio to experiment 1). Each of the three painted male colours was randomly assigned to one of three cages each day (Fig 2B). Behaviour was recorded as in experiment 1. This experimental design allowed us to 1) compare activity levels among painted and non-painted individuals to control for a possible effect of paint, and 2) to compare harassment rates received by painted and non-painted males directly, most specifically for blue-painted males, to determine whether they received similar harassment levels to unaltered males. Eight replicates (day) of experiment 2 were performed between June 23^rd^ and July 5^th^, 2005.

### Statistical Analysis

All analyses were performed in R 3.1.2 “Pumpkin Helmet” [32] using the mixed model glmer function of the package lme4. Our dependent variable was the total number of harassments received (or the total number of harassments given) by different types of male over the 30 minute observation period. For both experiments, we included the predictor variables cage and block as random factors (with cage nested within block) to control for, and partition, variation in male activity between days and cages. Given that the response was based on counts, we assumed a Poisson error variance. For experiment 1, our primary predictor of interest was the colour of the painted male, which was treated as a fixed factor. For experiment 2, the predictors of interest were whether the focal male was painted, and the colour of the painted male (focal or non-focal), which were both treated as fixed factors. Given the Poisson error we had assumed, the significance of fixed factors was evaluated through an analysis of deviance (log likelihood ratio tests, LRT). *Post hoc* tests for statistically significant main effects were conducted using the glht function of the multcomp package.

## Results

### Experiment 1

**Fig 3:**
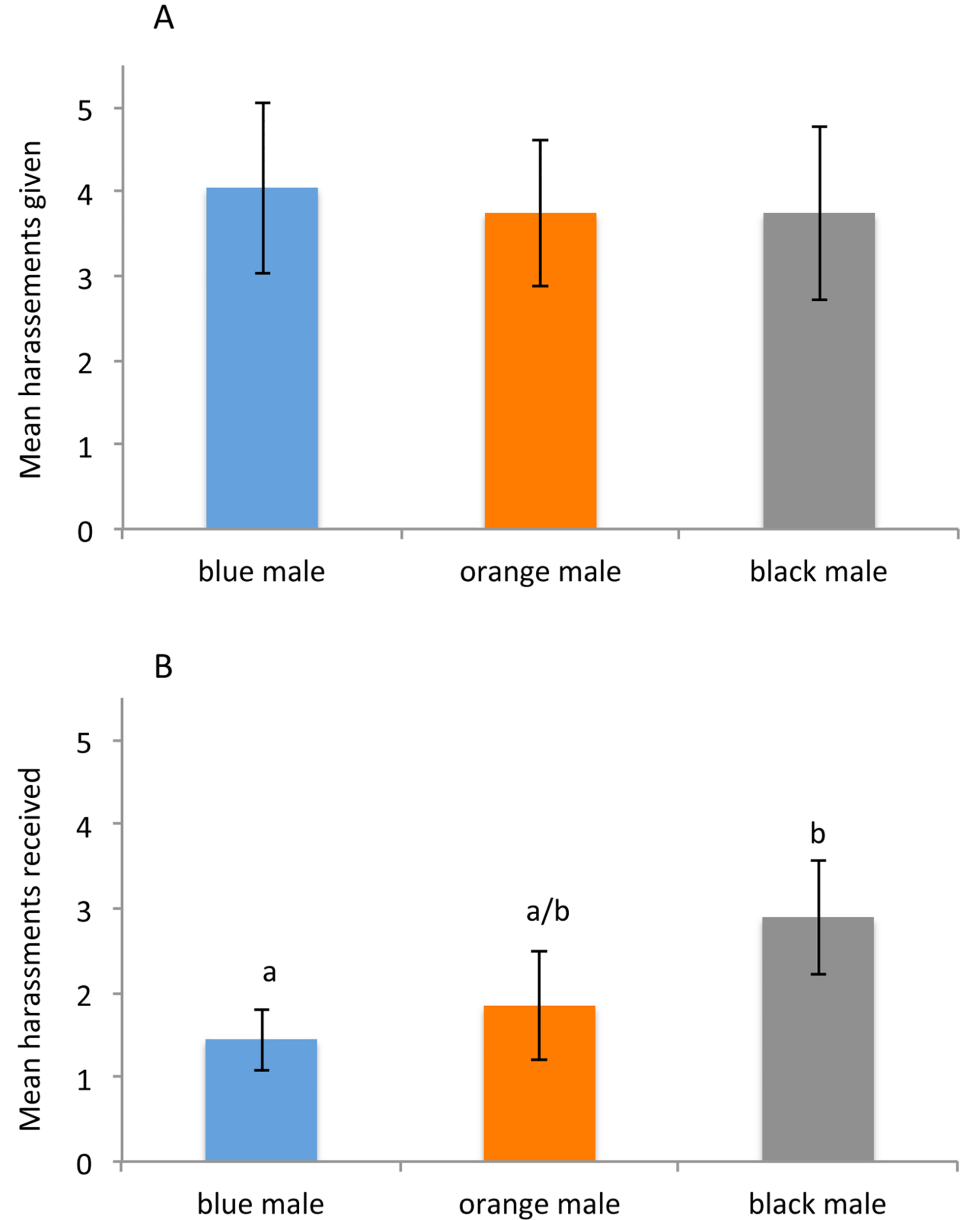
Comparison of harassment rates in males of *Nehalennia irene* painted different colors. Results of 30-minute behavioural observations over each of the *N = 20* replicates of experiment 1 (total of 20 cages, 236 observed harassments overall). A) Mean rates of harassment (± 1 SE) in which each male morph (blue, orange, black) was the instigator of an interaction. There was no significant difference among different male types in their harassment rates. B) Mean rates of harassments (± 1 SE) in which each of the male morphs was the recipient of harassment. Black-painted males were significantly more likely to be the recipients of harassment than blue- or orange-painted males (Tukey Contrasts multiple comparisons of means).

A total of 20 replicates (in 4 blocks) of experiment 1 were performed; over all these replicates a total of 236 harassments were observed. Fig 3 shows the rates of harrassment in experiment 1 for each type of male; Fig 3A shows the mean rates at which each type of individual was the giver of harassment, Fig 3B the mean rates at which the male form was the receiver of harassment. Our linear fixed-effect model found no difference in the total number of harassments per cage given by each of the three types of male morph (LRT comparing model with block/cage vs model with block/cage + colour X^2^ = 0.3091, df = 2, *P* = 0.8568). However, analysis of the total number of harassments per cage *received* by each of the three types of morph found a significant effect of colour on harassment (LRT comparing model with block/cage vs model with block/cage + colour X^2^ = 10.549, df = 2, *P* = 0.005122). Tukey Contrasts multiple comparisons of means indicated significantly higher harassment of black-painted males as compared to blue-painted males (*Z* = 3.128, *P*_BLUE-PAINT *VS.* BLACK-PAINT_ = 0.00497). while the harassment received by orange-painted males was not significantly different than either the harrassment rates of black- and blue- painted males (*P*_ORANGE-PAINT *VS.* BLACK-PAINT_ *=* 0.07193, *P*_ORANGE-PAINT *VS.* BLUE-PAINT_ *=* 0.57033).

### Experiment 2

**Fig 4:**
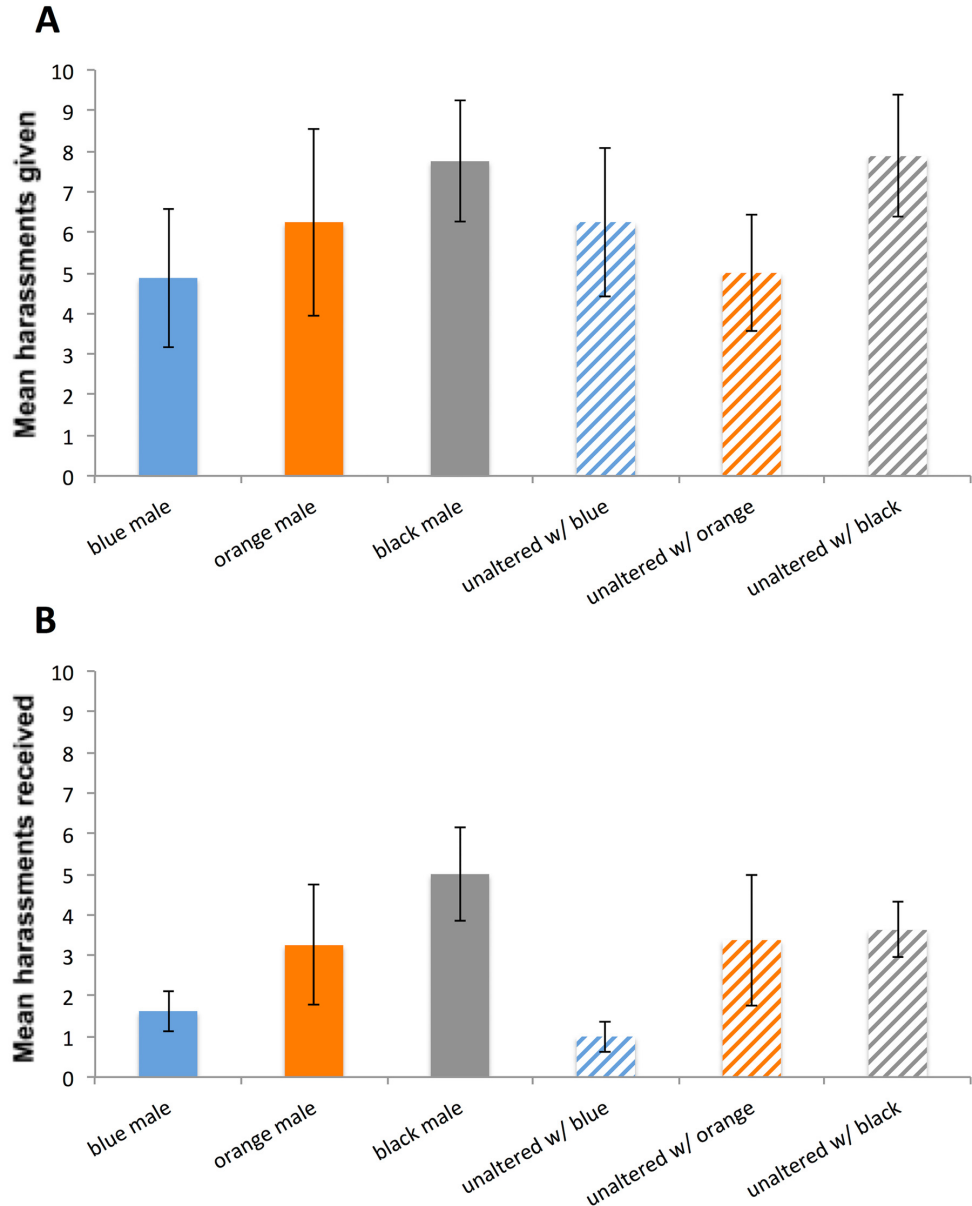
Comparison of harassment rates in painted and unpainted males of *Nehalennia irene*. Results of 30-minute behavioural observations over each treatment in the *N = 8* replicates of experiment 2, showing mean harassment rates (± 1 SE) for each type of painted male (solid-color columns) and the unpainted males placed in the cage with each of them (striped column of corresponding color). A total of 304 harrassments were observed. In A) the mean proportion of the harassments in which each type of male was the instigator of an interaction is shown; no significant difference was found among the different male types. In B) the mean number of interactions in which each type of male was the recipient of the harassment is presented. While there was no effect of paint *per se* (i.e., whether or not a male was painted did not directly influence the harassment it received), males in cages where painted males were black received a significantly higher rate of harassment than males in cages where painted males were blue (based on Tukey Contrasts multiple comparisons of means).

A total of 8 replicates of the block design for experiment 2 were performed; over all these replicates a total of 304 harrassments were observed. Considering painted and unpainted males together, there was no influence of painting per se on the propensity to give harassment (LRT comparing model with block/cage vs model with block/cage + painting X^2^ = 0.0132, df = 1, *P* = 0.90868, Fig 4A). There was no effect of paint colour on propensity to harass (LRT comparing model with block/cage vs with block/cage + paint colour X^2^ = 4.136, df = 2, *P* = 0.1263). Indeed, the full model with block/cage, paint colour and painting explained no significant additional variance compared to the model with just block/cage (X^2^ = 4.1517, df = 3, P = 0.2455).

When considering the rates at which males *received* harassment, there was no significant influence of painting (X^2^ = 1.5738, df = 1, *P* = 0.209661) but there was a signficant influence of colour (X^2^ = 9.6818, df = 2, *P* = 0.0079, Fig 4B); as predicted by the intraspecific aposematism hypothesis, males in cages with blue-painted males received significantly lower harassment than those in cages with black-painted males (Tukey Contrasts multiple comparisons of means: blue vs. black *Z* = 3.611, *P* = 0.000874), while the harassment received by orange-painted males was not significantly different than either the harrassment rates of black- and blue- painted males (*P*_ORANGE-PAINT *VS.* BLACK-PAINT_ *=* 0.213813, *P*_ORANGE-PAINT *VS.* BLUE-PAINT_ *=* 0.125620; Fig 4B). The full model with block/cage, paint colour and painting explained significant additional variance compared to the model with just block/cage (X^2^ = 11.258 df = 3 P = 0.01041), but this full model did not explain significant additional variance over and above the model with block/cage and paint colour alone (X = 1.5763, df = 1, P = 0.2093).

## Discussion

Here we have altered *N*. *irene* male coloration and observed the effect of this manipulation on harrassment by conspecific males. To our knowledge these experiments present the first explicit field tests of the intraspecific aposematism theory [13]. If conspicuous male coloration in *N*. *irene* functions as a warning signal to other males of their unprofitability as mates, then males bearing the typical male coloration ought to be harassed less than males with coloration similar to that of females. Our results indicate that this is indeed the case. The rates of harrassment of blue-painted males by other males was nearly half that of the rates of harrassment of black-painted males in both experiments. The lowered harassment received by blue-painted males indicates that male coloration may influence other males in the decision to approach them as possible mates. Black-painted males, appearing (at least to human eyes) more similar to females received higher rates of harassment.

Rates of harassment of orange painted males in our experiments were not significantly different from that of blue-painted or black-painted males. As orange is not a characteristic colour for this species, it may be that they were percieved neither as males and consistenty avoided, nor as females and consistently approached. As with more traditional warning signals, if the coloration of coenagrionid males functions as a signal of unprofitability, then displaying a distinct signal should help in avoiding confusion with profitable individuals [33–35]. Thus, those individuals bearing the coloration that serves as a badge of male identity (blue in this species) experienced the lowest rates of harassment. These results are in line with recent finds of Xu et al. [25] in their work on female-limited mimicry in damselflies: in that study, males painted both orange and pink (novel colour compared to the blue of males and male mimicking females and green females) experienced higher rates of harassment, causing the authors to suggest a first rule ‘if not blue, then female’ in the process of discerning males and female mimics.

Why might the colour blue serve as an effective ‘colour badge’ in this species? Warning colours are predominantly conspicuous; research has shown that conspicuous colours are easier to learn and remember [36], and that selection may have favoured conspicuous warning signals as ‘reliable’ signals, such that they are honest signals that profitable individuals cannot easily mimic [34,35]. Thus, if male colours serve as an intraspecific warning signal in some damselflies, it could be predicted that they would display signals with heightened conspicuousness in their specific habitat conditions. Schultz et al. [30] tested this, finding that male coloration among six damselfly species in the genus *Enallagma* (family Coenagrionidae) showed interspecific colour differences, with the colour of each species (blue or red) offering maximum contrast against natural backgrounds under the ambient light conditions when each of the species was most active. In the pond environments commonly inhabitated by *N*. *irene* and other coenagrionid damselflies, blue is an effective contrast, and is a common colour in these species.

Our results in experiment 2 offer some interesting nuances in comparison to our straighforward results in experiment 1. Here again there is an influence of colour of paint on harassments received, but no effect of whether a male was painted or not on harassment. Thus, harassment rates were lower for all males in the cage that contained blue-painted males compared to all males in cages with black-painted males. (Fig 4B). This may suggest a subtle effect of signal reliability on male behavior: in cages where painted males were blue, then all individuals carrying blue coloration were males, and thus potential mates were straightforward to determine. In cages where males were painted black, then the normal coloration of females is not as informative, since half of all individuals that are black are male, and half are female, making the conditions very confusing. If blue-painted males look like ‘typical’ males, one would expect that cages with all blue males (*i*.*e*. both painted and unaltered) would suffer lower levels of harassment because they all display the same ‘correct’ signal, thereby minimising confusion about male (and female) identity. This merits more study, but suggests a reason why there might be selection for a single, reliable signal of unprofitability.

Previous work on male response to conspecifics has focused predominantly on the coloration and morphology of female models. In a study intended to identify which visual/morphological cues male damselflies use in sex recognition, Gorb [20] presented male damselflies with male and female models with different body components interchanged or painted to look like the opposite sex. Gorb found that male *Coenagrion puella* tended not to show aggression to one another, and that females painted to look like males repelled conspecifics as effectively as male models.

Miller and Fincke [21] presented models to males of the species *Enallagma ebrium* to determine the responses that the female morph (andromorph or heteromorph—see [37–39]) elicited. They also investigated which components of female coloration are used by males in mate recognition. Females modified to look like males in their study received lowered sexual responses from males; likewise (with significant implications for our present study) males modified to look like females—through blackening of conspicuous abdominal coloration—experienced a considerable increase in sexual responses by other males.

In recent work on the effectiveness of male mimicry in female damselflies, Xu et al. [25] showed that males can develop decision rules in identifying potential mates, using colour, pattern and potentially other morphological attributes to identify males, and both mimetic and non-mimetic females. Likewise, Gorb’s [20] results indicated that, while male-like thoracic coloration did diminish male response to females, abdomen and wing coloration function as the primary cues used by males to identify females, and thus multiple signal components can be used in selecting potential mates. We altered only abdominal coloration, and not thoracic colour, so our black-painted males were at best imperfect female mimics; it should be noted that in all our experiments, the majority of mating attempts were still directed at females. Thus, ours was a somewhat conservative test of the intraspecific aposematism hypothesis, as we altered only a single component of coloration, and still found a significant difference is male response to other males.

While our results do not in themselves demonstrate that sexual colour dimorphism originally evolved as an intra-specific warning signal, we have demonstrated that female-like males are harrassed at higher rates than males with conspicuous coloration, a necessary (but not sufficient) condition if the anti-harassment aposematism hypothesis is correct. Further studies of this system should focus on the implications of increased harassment for male survivorship and reproductive sucess. Córdoba-Aguilar [40] found that females of the territorial damselfly species *Hetaerina americana* suffered reduced mating opportunities and survival in conditions of increased male harassment; females reallocated fat reserves and immune response activity to early egg production in response to this harassment. For *N*. *irene* and other non-territorial species, conspicuous coloration in males may be a way to avoid harassment from conspecific males.

## References

[1] West-Eberhard MJ. Sexual selection, social competition, and speciation. Quart. Rev. Biol. 1983;58: 155–183.

[2] Andersson M. Sexual selection Princeton: Princeton University Press; 1994.

[3] Corbet PS. Dragonflies: Behaviour and Ecology of Odonata. Ithaca: Comstock Publishing Associates; 1999.

[4] Gibbons DW, Pain D. The influence of river flow-rate on the breeding behavior of *Calopteryx* damselflies. J. Anim. Ecol. 1992;61: 283–289.

[5] Grether GF. Intrasexual competition alone favors a sexually dimorphic ornament in the rubyspot damselfly *Hetaerina americana* . Evolution 1996;50: 1949–1957. 28565578 10.1111/j.1558-5646.1996.tb03582.x

[6] Hooper RE, Tsubaki Y, Siva-Jothy MT. Expression of a costly, plastic secondary sexual trait is correlated with age and condition in a damselfly with two male morphs. Physiol. Entom. 1999;24: 364–369.

[7] Siva-Jothy MT. A mechanistic link between parasite resistance and expression of a sexually selected trait in a damselfly. Proc. Roy. Soc. B 2000;267: 2523–2527. 10.1098/rspb.2000.1315 PMC1690847 11197129

[8] Emlen ST, Oring LW. Ecology, sexual selection, and the evolution of mating systems. Science 1977;197: 215–233. 327542 10.1126/science.327542

[9] Ubukata H. Mating system of the dragonfly *Cordulia aenea amurensis* Selys and a model of mate searching and territorial behaviour in Odonata In: Brown JL, Ito Y, Kiawa J, editors. Animal Societies: theories and facts, Tokyo: Japan Science Society Press; 1987.

[10] Conrad KF, Pritchard G. An ecological classification of odonate mating systems–the natural influence of natural, intersexual and intrasexual selection on males. Biol. J. Linn. Soc. 1992;45: 255−269.

[11] Battin T. The odonate mating system, communication, and sexual selection: a review. Boll. Zool. 1993;60: 353–360.

[12] Corbett PS. Dragonflies: Behaviour and Ecology of Odonata. Ithaca, New York: Comstock Publishing Associates; 1999.

[13] Sherratt TN, Forbes MR. Sexual difference in coloration of Coenagrionid damselflies (Odonata): a case of intraspecific aposematism? Anim. Behav. 2001;62: 653–659.

[14] Papaj DR, Newsom GM. A within-species warning function for an aposematic signal. Proc. Roy. Soc. B 2005;272: 2519–2523. 10.1098/rspb.2005.3186 PMC1599780 16271978

[15] Poulton EB. The Colours of Animals: their Meaning and Use especially considered in the case of Insects London: Kegan Paul, Trench, Trübner & Co. Ltd; 1890.

[16] Catling PM, Brownell VR. Damselflies and Dragonflies (Odonata) of Ontario: Resource Guide and Annotated List. Toronto: Toronto Entomological Association; 2000.

[17] Lam E. Damselflies of the northeast: A guide to the species of eastern Canada and the northeastern United States New York: Biodiversity Books; 2004.

[18] Van Gossum H, Beirinckx K, Forbes MR, Sherratt TN. Reproductive interference between *Nehalennia* damselfly species. Ecoscience 2007;14: 1–7.

[19] Van Gossum H, Beirinckx K, Forbes MR, Sherratt TN. Continental and seasonal variation in female morph frequencies of the damselfly, *Nehalennia irene* . Biol. J. Linn. Soc. 2007;90: 501–508.

[20] Gorb SN. Visual cues in mate recognition by males of the damselfly Coenagrion puella (L.) (Odonata: Coenagronidae). J. Ins. Behav. 1998;11: 73–92.

[21] Miller MN, Fincke OM. Cues for mate recognition and the effect of prior experience on mate recognition in *Enallagma* damselflies. J. Ins. Behav. 1999;12: 801–814.

[22] Andrés JA, Sanchez-Guillen RA, Cordero Rivera A. Evolution of female colour polymorphism in damselflies: testing the hypotheses. Anim. Behav. 2002;63: 677–685.

[23] Iserbyt A, Van Gossum H. Show your true colour: cues for male mate preference in an intra-specific mimicry system. Ecol. Ent. 2011;36: 544–548.

[24] Cordero Rivera A, Andrés JA. Estimating female morph frequencies and male mate preferences of polychromatic damselflies: a cautionary note. Anim. Behav. 2001;61: F1–F6.

[25] Xu M, Cerreta AL, Schultz TD, Fincke OM. Selective use of multiple cues by males reflects a decision rule for sex discrimination in a sexually mimetic damselfly. Anim. Behav. 2014;92: 9–18.

[26] Bybee SM, Johnson K\K, Gering EJ, Whiting MF, Crandall KA. All the better to see you with: a review of odonate color vision with transcriptomic insight into the odonate eye. Org. Div. & Evolution, 2012;12, 241e250.

[27] Lavoie-Dornik J, Pilon J-G, Gogala M, Li MA. Étude électrophysiologique de la croissance de l’oeil composé de *Enallagma cyathigerum* (Charpentier) et *E*. *clausum* Morse (Zygoptera: Coenagrionidae). Odonatologica 1988;17: 337–355.

[28] Yang EC, Osorio D. Spectral sensitivities of photoreceptors and lamina monopolar cells in the dragonfly *Hemicordulia tau* . J. Comp. Phys. A 1991;169: 663–669.

[29] Briscoe AD, Chittka L. The evolution of color vision in insects. Ann. Rev. Ent. 2001;46: 471–510. 10.1146/annurev.ento.46.1.471 11112177

[30] Schultz TD, Anderson CN, Symes LB. The conspicuousness of colour cues in male pond damselflies depends on ambient light and visual system. Anim. Behav. 2008;76: 1357–1364.

[31] Van Gossum H, Bots J, Van Heusden J, Hammers M, Huyghe K, Morehouse NI. Reflectance spectra and mating patterns support intraspecific mimicry in the colour polymorphic damselfly *Ischnura elegans* . Evol. Ecol. 2011;25: 139–154.

[32] RCoreTeam. R: A language and environment for statistical computing R Foundation for Statistical Computing, Vienna, Austria 2013 Available: http://www.R-project.org/.

[33] Fisher RA. The Genetical Theory of Natural Selection, Oxford: Oxford University Press; 1930.

[34] Sherratt TN. The coevolution of warning signals. Proc. Roy. Soc. B 2002;269: 741–746. 10.1098/rspb.2001.1944 PMC1690947 11934367

[35] Sherratt TN, Beatty CD. The evolution of warning signals as reliable indicators of prey defense. Am. Nat. 2003;162: 377–389. 14582002 10.1086/378047

[36] Ruxton GD, Sherratt TN, Speed MP. Avoiding attack: The Evolutionary Ecology of Crypsis, Warning Signals and Mimicry. Oxford: Oxford University Press; 2004.

[37] Robertson HM. Female dimorphism and mating behaviour in a damselfly, *Ischnura ramburi*: females mimicking males. Anim. Behav. 1985;33: 805–809.

[38] Fincke OM. Female dimorphism in damselflies: failure to reject the null hypothesis. Anim. Behav. 1994;47: 1249–1266.

[39] Cordero Rivera A, Santolamazza Carbone S, Utzeri C. Mating opportunities and mating costs are reduced in androchrome female damselflies, *Ischnura elegans* (Odonata). Anim. Behav. 1998;55: 185–197. 9480685 10.1006/anbe.1997.0603

[40] Córdoba-Aguilar A. A female evolutionary response when survival is at risk: male harassment mediates early reallocation of resources to increase egg number and size. Behav. Ecol. & Sociobiol. 2009;63:751–763.

